# Highly stable *meso*-diaminopimelate dehydrogenase from an *Ureibacillus thermosphaericus *strain A1 isolated from a Japanese compost: purification, characterization and sequencing

**DOI:** 10.1186/2191-0855-1-43

**Published:** 2011-11-25

**Authors:** Hironaga Akita, Yasuhiro Fujino, Katsumi Doi, Toshihisa Ohshima

**Affiliations:** 1 Applied Molecular Microbiology and Biomass Chemistry, Bioscience and Biotechnology, Faculty of Agriculture, Kyushu University, 6-10-1 Hakozaki, Higashi-ku, Fukuoka 812-8581, Japan; 2 Center for Research and Advancement in Higer Education, Kyushu University, 744 Motooka, Nishi-ku, Fukuoka 819-0395, Japan; 3 Microbial Genetic Division, Institute of Genetic Resources, Faculty of Agriculture, Kyushu University, 6-10-1 Hakozaki, Higashi-ku, Fukuoka 812-8581, Japan

**Keywords:** *meso*-Diaminopimelate dehydrogenase, *Ureibacillus thermosphaericus*, Thermostable amino acid dehydrogenase, Purification and characterization

## Abstract

We screened various thermophiles for *meso*-diaminopimelate dehydrogenase (*meso*-DAPDH, EC 1.4.1.16), which catalyzes the NAD(P)-dependent oxidative deamination of *meso*-diaminopimelate, and found the enzyme in a thermophilic bacterium isolated from compost in Japan. The bacterium grew well aerobically at around 55°C and was identified as *Ureibacillus thermosphaericus *strain A1. We purified the enzyme about 47-fold to homogeneity from crude cell extract using five successive purification steps. The molecular mass of the purified protein was about 80 kDa, and the molecule consists of a homodimer with the subunit molecular mass of about 40 kDa. The optimum pH and temperature for the catalytic activity of the enzyme are about 10.5 and 65°C, respectively. The enzyme is highly selective for *meso*-diaminopimelate as the electron donor, and NADP but not NAD can serve as the electron acceptor. The *K*_m _values for *meso*-diaminopimelate and NADP at 50°C and pH 10.5 are 1.6 mM and 0.13 mM, respectively. The nucleotide sequence of this *meso*-DAPDH gene encodes a 326-amino acid peptide. When the gene was cloned and overexpressed in *Escherichia coli *Rosetta (DE3), the specific activity in the crude extract of the recombinant cells was about 18.0-fold higher than in the extract from *U. thermosphaericus *strain A1. This made more rapid and simpler purification of the enzyme possible.

## Introduction

*meso*-Diaminopimelate dehydrogenase (*meso*-2,6-D-diaminopimerate dehydrogenase, *meso*-DAPDH, EC 1.4.1.16) catalyzes the NADP-dependent oxidative deamination of *meso*-2,6-diaminopimelate (*meso*-DAP) to produce L-2-amino-6-oxopimelate (L-2-amino-6-oxoheptanedioate). This enzyme is the only known NAD(P)-dependent dehydrogenase able to stereoselectively act on the D-configuration of *meso*-DAP. It has been identified in several bacteria, and is known to function in L-lysine biosynthesis in *Bacillus sphaericus *(Misono et al. 1979) and *Corynebacterium glutamicum *(Misono et al. 1986a). In addition, it has been purified to homogeneity from *B. sphaericus *(Misono and Soda 1980) and *Brevibacterium *sp. (Misono et al. 1986b), and has been characterized enzymologically. The *meso*-DAPDH genes from *C. glutamicum *(Ishino et al. 1988) and *B. sphaericus *(Sakamoto et al. 2001) have been sequenced, and were found to be highly similar to one another. The *C. glutamicum *gene has been expressed in *Escherichia coli *cells (Reddy et al. 1996), and the three-dimensional structures of the enzyme-NADP complex (Scapin et al. 1996), the enzyme-substrate complex and an enzyme-NADP-inhibitor complex (Scapin et al. 1998) have been solved for the *C. glutamicum *enzyme and refined to 2.2 Å resolution.

An NADP-dependent, highly stereoselective D-amino acid dehydrogenase was also prepared through mutation of *C. glutamicum meso*-DAPDH using both rational and random mutagenesis (Vedha et al. 2006). The mutant enzyme is potentially useful for the production of D-amino acids via the reductive amination of the corresponding 2-oxo acid with ammonia. However, the mutant enzyme is not necessary stable enough to use for a long term and under various conditions. Thus, more stable *meso*-DAPDH and its mutant enzyme have been required. We had looked for the enzyme in thermophiles by database and the activity analyses, but were not able to find the homologous gene in sequence to that of *C. glutamicum meso*-DAPDH in thermophiles. Thus, we had started the screening of stable *meso*-DAPDH by detection of the enzyme activity in many strains of thermophiles stocked as type cultures and isolated from soils and composts, and found the activity in an aerobically well-grown thermophile from a compost. Just recently, *meso*-DAPDH in a thermophilic bacterium, *Clostridium thermocellum *was found and the enzymological properties were reported with emphasizing to show the presence of *meso*-DAPDH pathway in as well as a succinyl and acetyl-DAP pathway (Hudson et al, 2011). In the present study, the thermophile of *meso*-DAPDH producer isolated from compost was identified to be *Ureibacillus thermosphaericus *strain A1. The enzyme was then purified from the thermophile and characterized as a thermostable *meso*-DAPDH, and the gene was sequenced.

## Materials and methods

### Materials

An illustra bacteria genomicPrep Mini Spin Kit was purchased from GE Healthcare (Buckinghamshire, UK). A HiYield™ Plasmid Mini Kit was from RBC Bioscience (Taipei, Taiwan). A QIAquick Gel Extraction Kit was from QIAGEN (Hilden, Germany). Restriction endonucleases were purchased from Takara Bio (Shiga, Japan) and Toyobo (Osaka, Japan). Butyl Sepharose™ 4 Fast Flow was from GE Healthcare. DEAE-Toyopearl M-650 was from Tosoh (Tokyo, Japan). Amicon Ultra-15 was from Millipore (Billerica, MA, USA). INT (2-(4-Iodophenyl)-3-(4-nitrophenyl)-5-phenyl-2H-tetrazolium chloride) and 1-Methoxy PMS (1-Methoxy-5-methylphenazinium methyl sulfate) were from Dojindo (Kumamoto, Japan). All other chemicals were reagent grade.

### Screening for *meso-*DAPDH in thermophiles and the growth conditions for *U. thermosphaericus*

We isolated thermophiles from a variety of soils, sea sands, composts and mud from hot springs at 50-70°C using medium containing 0.5% polypeptone-S (Nihonseiyaku, Tokyo), 0.2% meat extract (Wako Pure Chemical Industries, Osaka), 0.35% NaCl and 2% agar (pH 7.2 with KOH). After cultivation, the cells were collected by centrifugation (8,000 × *g *for 15 min at 4°C), and the terrestrial microorganisms were washed twice with 0.85% NaCl, while the marine microorganisms were washed with 3% NaCl. The cells were then suspended with a small amount of 10 mM potassium phosphate buffer (pH 7.2) containing 10% glycerol and stored at -80°C until used. A *meso*-DAPDH producing thermophile, *U. thermosphaericus *strain A1 was aerobically cultured over night at 50°C in the liquid medium described above on a reciprocating rotor (250 rpm). The screening procedure for detection of *meso*-DAPDH entailed native polyacrylamide gel electrophoresis (native-PAGE) followed by activity staining at 50°C with a mixture containing 300 mM potassium phosphate buffer (pH 8.0), 50 mM *meso*-DAP, 0.1 mM INT, 0.04 mM 1-Methoxy PMS and 2.5 mM NADP until a red band of sufficient intensity had developed. Enzyme activity was then assessed spectrophotometrically as described below in the "Enzyme assay" section.

### 16S rRNA gene amplification and sequencing

Genomic DNA was extracted from isolated bacteria using an illustra bacteria genomic Prep Mini Spin Kit and then used as the template for 16S rRNA gene amplification. DNA fragments were amplified by polymerase chain reaction (PCR) using the universal primers 27f (5'-AGAGTTTGATCMTGGCTCAG-3') and 1492r (5'-TACGGYTACCTTGTTACGACTT-3'). PCR mixture contained 10× Ex *Taq *buffer, 0.2 mM dNTP mixture, 100 ng of DNA template, 1.0 μM primers 27f and 1492r, and 1.25 U of Ex *Taq *DNA polymerase (Takara Bio) in a final volume of 50 μl. The PCR protocol entailed a 30 s denaturation at 98°C, followed by 30 cycles of 98°C for 30 s, 51°C for 30 s and 72°C for 1.7 min and a final extension at 72°C for 10 min in TProfessional 96 Gradient (Biometra, Göttingen, Germany). The amplified PCR products were purified using Wizard^® ^SV Gel and a PCR Clean-up System (Promega, WI, USA) to remove unconsumed dNTPs and primers and then directly sequenced using a BigDye^® ^Terminator v3.1 Cycle Sequencing Kit (Applied Biosystems, CA, USA) on a 3130 Genetic Analyzer.

### Phylogenetic analysis of the 16S rRNA gene sequences

The 16S rRNA gene sequences from the bacteria isolated in this study were aligned and clustered against those of the genus *Ureibacillus *(Fortina et al 2001), which was available from GenBank.

### Enzyme assay and protein determination

The rate of NADP-dependent oxidative deamination of *meso*-DAP was spectrophotometrically determined at 50°C. The standard reaction mixture (total volume: 1.00 ml) contained 200 mM carbonate-KOH (pH 10.5), 10 mM *meso*-DAP, 1.25 mM NADP and enzyme. The mixture without the coenzyme (NADP) was pre-incubated at 50°C for about 3 min in a cuvette with a 1.0-cm light path. The reaction was then started by adding 25 mM NADP (50 μl), which had also been pre-incubated at 50°C. The increase in absorbance accompanied by the formation of NADPH was monitored at 340 nm (an extinction coefficient = 6.22 mM^-1 ^cm^-1^). One unit of enzyme was defined as the amount catalyzing the formation of 1 μmol of NADPH/min at 50°C during *meso*-DAP oxidation. The protein concentration was determined by the method of Bradford (1976) using bovine serum albumin as the standard.

### Purification of *meso*-DAPDH from *U. thermosphaericus *cells

All steps in the purification procedure were carried out at a room temperature, using 50 mM potassium phosphate buffer (pH 7.2) as the standard buffer. *U. thermosphaericus *cells (5.12 g, wet weight) suspended in about 20 ml of standard buffer were disrupted by sonication (UD-201; Tomy Seiko, Tokyo), which entailed five cycles of 60-s pulses (50 W) followed by a 60-s rest on ice. Thereafter, any remaining intact cells and the cell debris were removed by centrifugation (27,500 × *g *for 20 min at 4°C), and the resultant supernatant was used as the crude extract. Ammonium sulfate was added to the crude extract to 80% saturation, and the precipitate obtained by centrifugation (27,500 × *g *for 20 min at 4°C) was dissolved in standard buffer supplemented with 1 M (NH_4_)_2_SO_4_. The resultant solution was then applied to a Butyl Sepharose™ 4 Fast Flow column (6.1 × 10.0 cm) equilibrated with standard buffer supplemented with 1 M (NH_4_)_2_SO_4_. The column was then washed with the same supplemented buffer, and the enzyme was eluted with a linear 1.0 to 0 M (NH_4_)_2_SO_4 _gradient in the same buffer. The active fractions were pooled and dialyzed against the standard buffer, after which the dialyzate was loaded onto a DEAE-Toyopearl 650 M column (6.1 × 5.0 cm) equilibrated with the standard buffer. After washing the column with standard buffer, the enzyme was eluted with a linear 0 to 0.5 M NaCl gradient in the same buffer. The active fractions were again pooled and dialyzed against the standard buffer, and preparative slab PAGE was carried out according to the method of Ohshima and Ishida (1992). Finally, the enzyme was extracted from the gel pieces using the standard buffer and Amicon Ultra-15, and the resultant enzyme solution was used for experimentation.

### PAGE and molecular mass determination

Native-PAGE was carried out at 4°C on a 7.5% polyacrylamide gel using the method of Davis (1964). The protein was then stained using 0.025% Coomassie brilliant blue R-250 in 50% methanol and 10% acetate. In addition, active staining was performed at 50°C using a mixture containing 300 mM potassium phosphate buffer (pH 8.0), 50 mM *meso*-DAP, 0.1 mM INT, 0.04 mM 1-Methoxy PMS and 1.25 mM NADP until a red band of sufficient intensity was visible.

Sodium dodecyl sulfate (SDS)-PAGE was carried out on a 10% polyacrylamide gel using the method of Laemmli (1970). Precision Plus protein standards (Bio-Rad Laboratories, CA, USA) were used as the molecular mass standards. The protein sample was boiled for 5 min in 10 mM Tris-HCl buffer (pH 7.0) containing 1% SDS and 1% 2-mercaptoethanol. Protein bands were visualized by staining with 0.025% Coomassie brilliant blue R-250 in 50% methanol and 10% acetate.

The molecular mass of the native enzyme was determined by gel filtration column chromatography using a Superdex 200 pg column (2.6 × 60 cm). Ferritin (440 kDa), aldolase (158 kDa), conalbumin (75 kDa), ovalbumin (43 kDa) and α-chymotrypsinogen (25 kDa) served as molecular standards (GE Healthcare).

### Determination of kinetic parameters

The Michaelis constant (*K*_m_) was determined from double-reciprocal plots of the initial rate data using *meso*-DAP as the electron donor and NADP as the electron acceptor at 50°C.

### N-Terminal amino acid sequence analysis

The N-terminal amino acid sequence of the isolated enzyme was analyzed using an automated Edman degradation protein sequencer. The phenylthiohydantoin derivatives were separated and identified using a Shimadzu PPSQ-10 protein sequencer (Shimadzu, Kyoto, Japan).

### Purification of genomic DNA

To obtain genomic DNA for *meso*-DAPDH gene sequencing, genomic DNA was prepared from *U. thermosphaericus *as follows. *U. thermosphaericus *cells (about 1 g, wet weight) were suspended in 10 ml of 50 mM Tris-HCl buffer (pH 8.0) containing 50 mM EDTA, 10 mg of lysozyme per ml and 100 mg of proteinase K (Nacalai Tesque, Kyoto) per ml, and incubated for 3 h at 37°C. This was followed by addition of 10% SDS (1.2 ml) and incubation for 15 min at 65°C, after which CTAB-NaCl solution (1.5 ml of 100 mM Tris-HCl (pH 9.0), 10 mM EDTA, 1.4 M NaCl and 2.0% cetyltrimethylammonium bromide solution) was added, and the cells were incubated for an additional 15 min at 65°C. The proteins in the mixture were then extracted several times with phenol-chloroform, and the genomic DNA was precipitated first with 2.5 volumes of 100% ethanol supplemented with 60 μl of 3 M sodium acetate buffer (pH 5.2), and then with 2.5 volumes of 70% ethanol for demineralization. To remove any contaminating RNA, RNase was then added to the solution and incubated for 3 h at 37°C.

### Screening for *meso*-DAPDH gene

For clonining of the *meso*-DAPDH gene from *U. thermosphaericus*, a homology search was carried out among the related strains using the NCBI BLAST program. Based on the highly-conserved regions of *meso*-DAPDH genes, a set of degenerated primers (forward: 5'-GGRATYGTMGGWTAYGGRAAY-3' [where M is A or C; R is A or G; W is A or T and Y is C or T], reverse: 5'-RACRAAWCCKCCRTGWGG-3' [where K is G or T; R is A or G and W is A or T]) were designed for the partial amplification of *meso*-DAPDH gene from *U. thermosphaericus*. PCR was performed by 30 cycles of 98°C for 30 s, 51°C for 30 s and 72°C for 50 sec with Ex *Taq *DNA polymerase. The amplified PCR products were purified and directly sequenced. The complete sequence of the *meso*-DAPDH gene was obtained using an *in vitro *Cloning Kit (Takara Bio) according to the manufacturer's instructions.

### Sequence analysis

**Figure 1:**
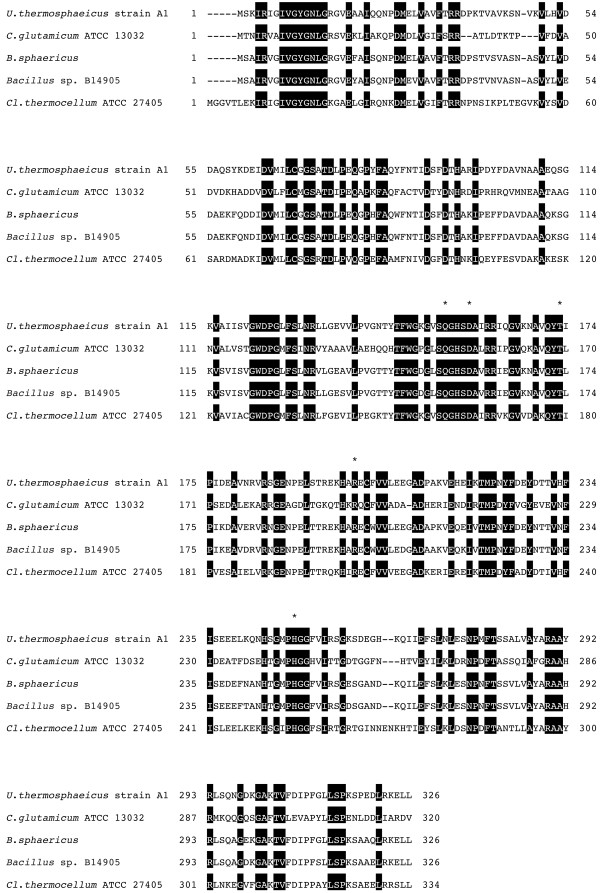
**Multiple sequence alignment of *meso*-DAPDHs**. The accession numbers of the aligned sequences are *C. glutamicum *ATCC13032 (YP_226858), *B. sphaericus *(BAB07799), *Bacillus *sp. B14905 (ZP_01724569), *Cl. thermocellum *ATCC27405 (ABN52156). Amino acid residues mutated for the creation of D-amino acid.

The sequence obtained as described above was identified by using the NCBI BLAST program to run similarity searches against other sequences from available databases. The sequences were then aligned using ClustalW (Larkin et al. 2007), and multiple sequence alignment of *meso*-DAPDHs (Figure 1) was generated.

### Expression of *meso*-DAPDH gene and purification of the gene product

*meso*-DAPDH gene was amplified using KOD -plus- DNA polymerase (Toyobo) with primers 5'-CACCATGAGTAAAATTAGAATTGGG-3' (forward) and 5'-TAAAAGTTCTTTTCTTAAATCTTCTGGAG-3' (reverse), and then cloned using a Champion™ pET 101 Directional TOPO Expression Kit (invitrogen, CA, USA), yielding the expression plasmid pET101/DAPDH. *E. coli *Rosetta (DE3) cells were transformed with pET101/DAPDH, after which the transformants were grown in LB medium (1% Triptone, 1% NaCl and 0.5% yeast extract) containing 0.01% ampicillin at 37°C. After 6 h of cultivation, IPTG (1 mM) was added, and the cultivation was continued for an additional 2 h at 37°C. The cells were then collected by centrifugation (8,000 × *g *for 15 min at 4°C), resuspended in standard buffer, and disrupted by sonication as described above. After removing the debris by centrifugation (27,500 × *g *for 20 min at 4°C), the supernatant was incubated for 30 min at 50°C (heat-treatment) and centrifuged (27,500 × *g *for 20 min at 4°C) again to remove the unwanted proteins derived from the bacteria. The resultant solution was loaded onto a Chelating Sepharose™ Fast Flow column (6.1 × 6.0 cm) (GE Healthcare) previously equilibrated with buffer containing 50 mM NiSO_4_, 20 mM Tris-HCl (pH 7.9), 500 mM NaCl and 5 mM imidazole. The column was then washed with washing buffer (20 mM Tris-HCl (pH 7.9), 500 mM NaCl, 60 mM imidazole), and the adsorbed proteins were eluted with the elution buffer (20 mM Tris-HCl (pH 7.9), 500 mM NaCl and 1 M imidazole). The active fractions were pooled and dialyzed against the standard buffer.

## Results

### Screening for *meso*-DAPDH in thermophiles

When we started this study about 2 years ago, there was no report on *meso*-DAPDH from thermophile as the producer of more stable *meso*-DAPDH. Thus, we screened more than 100 strains of aerobically grown thermophiles isolated from various soil samples by incubation at 50°C and 70°C, and detected *meso*-DAPDH activity in a thermophile isolated from compost obtained from Munakata City in Fukuoka Prefecture, Japan. When cultured in liquid medium, the thermophilic strain grew at temperatures between 37°C and 55°C, but not at 60°C, and maximum growth was achieved at 50°C. The crude extract from the cells included a high level of *meso*-DAPDH activity when NADP served as a coenzyme. To identify the thermophile, we determined the sequence of the 16S rRNA gene (1425 bp; accession number: AB671590), which was found to be completely identical to that of *U. thermosphaeicus* (accession number: X90640). Thus, the isolated strain was identified as *U. thermosphaeicus* strain A1 and deposited to Biological Resource Center (NBRC), National Institute of Technology and Evaluation (strain number: NBRC 108682).

### Purification of *meso*-DAPDH from the *U. thermosphaeicus*

**Figure 2:**
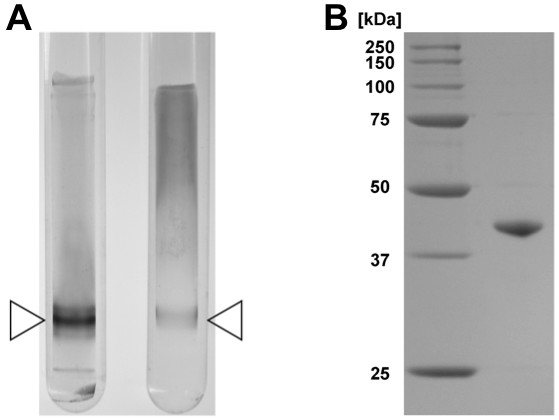
**PAGE of purified *meso-*DAPDH from *U. thermosphaeicus *strain A1**. A: Native PAGE of the purified enzyme. The purified enzyme was applied to a 7.5% acrylamide gel. The left and right lanes show the patterns of protein and activity staining, respectively. The triangle indicates the position of the bands. B: SDS-PAGE of the purified enzyme. The purified enzyme was applied to a SDS-PAGE on a 10% acrylamide gel. The left and right lanes show the positions of the molecular marker proteins and the purified enzyme.

We collected 5.14 g (wet weight) of *U. thermosphaeicus *cells from 500 ml of culture medium, and then obtained about 260 mg of soluble protein from the crude cell extract after sonication and centrifugation. The purified enzyme was then isolated using the five-step procedure described in the materials and methods. The purified *meso*-DAPDH migrated as a single band on both native PAGE (Figure 2A) and SDS-PAGE (Figure 2B), indicating the enzyme had been purified to homogeneity (Table 1). At the final step, the enzyme was purified about 47-fold, with an overall yield of about 4.69%.

**Table 1:** Purification of NADP-dependent *meso-*DAPDH from *U. thermosphaeicus *

**Purification step**^**a**^	Total protein	Total activity	Specific activity	Yield
	mg	units	units/mg	%
Crude extract	264	46.6	0.177	100
80% Ammonium sulfate	162	44.5	0.274	95.5
Butyl Sepharose™ 4 Fast Flow column	15.1	15.7	1.04	33.7
DEAE-Toyopearl 650M column	2.90	5.01	1.73	10.8
Preparative slab PAGE	0.264	2.19	8.28	4.69

a) The cells were obtained from a 500 ml-medium.

### Molecular mass and subunit structure

Following native-PAGE, the molecular mass of the *U. thermosphaeicus meso*-DAPDH was determined to be about 80 kDa using Superdex 200 pg column gel filtration chromatography (data not shown). SDS-PAGE of the enzyme showed one major band of 40 kDa (Figure 2B), suggesting that the enzyme is composed of two identical subunits.

### Effects of pH, temperature and various chemicals on enzyme activity and stability

**Figure 3:**
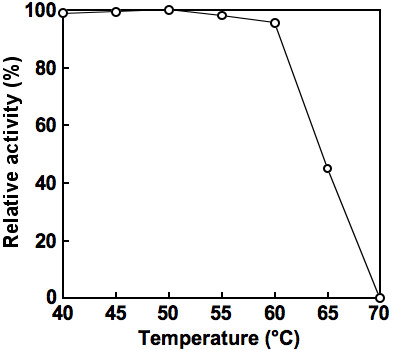
**Effect of temperature on the activity of NADP-dependent *meso-*DAPDH from *U. thermosphaericus *strain A1**. After the enzyme (in 10 mM potassium phosphate buffer, pH 7.2) was incubated for 30 min at each temperature, the residual activity was determined using the standard assay at 50°C.

The effect of pH on the oxidative deamination of *meso*-DAP was determined by assessing the enzyme activity at various pHs. At a temperature of 50°C, the optimum pH was about 10.5. When the temperature dependence of the catalytic activity at pH 10.5 was examined, maximum activity was observed at around 65°C. Moreover, when the enzyme was incubated for 30 min at various temperatures in the standard buffer at pH 7.2, no activity was lost at temperatures below 60°C, while half of the activity was lost at 65°C (Figure 3). When the effect of pH on the stability of the enzyme was evaluated based on the activity remaining after incubation at 50°C for 30 min, no loss of activity was observed at pHs between 5.0 and 11.0 (data not shown).

### Substrate and coenzyme specificities

Examination of the substrate specificity of the oxidative deamination of amino acids revealed that *meso*-DAP was the only effective substrate in presence of NADP. The enzyme was highly specific for *meso*-DAP as the electron donor, and the following amino acids were inert: DL-2-aminopimelate, D-glutamate, L-glutamate, D-aspartate, L-aspartate, D-alanine, L-alanine, D-valine, L-valine, D-lysine, L-lysine, D-phenylalanine, L-phenylalanine, D-leucine, L-leucine, D-threonine, L-threonine, D-serine, L-serine, D-tryptophan, L-tryptophan, D-cysteine, L-cysteine, D-histidine, L-histidine, D-methionine, D-arginine, D-proline, D-asparagine, D-glutamine, D-isoleucine and D-ornithine. Moreover, the enzyme exclusively used NADP as the coenzyme (electron acceptor) for the oxidative deamination of *meso*-DAP; NAD was inert.

### Inhibitors

The enzyme was completely inhibited by both 0.1 mM *p*-chloromercuribenzoate and HgCl_2_, which are typical inhibitors of SH enzymes. Thioglycollate (5 mM), L-cysteine (5 mM) and Cu^2+ ^(1 mM) also strongly inhibited the enzyme. Several other metal ions (1 mM), including Zn^2+^, Co^2+ ^and Ni^2+ ^were not inhibitory, nor did 1 mM D-lysine, L-lysine, EDTA, α,α'-dipyridyl, NaN_3 _or iodoacetic acid affect the oxidative deamination of *meso*-DAP.

### Kinetic studies

Analysis of the initial velocity of the oxidative deamination of *meso*-DAP in the presence of NADP yielded typical Michaelis-Menten kinetics. As determined from Lineweaver-Burk plots, the apparent *K*_m _values at 50°C in cabonate-KOH buffer (pH 10.5) were 1.6 mM and 0.13 mM for *meso*-DAP and NADP, respectively.

### N-Terminal amino acid sequencing

Using an automated Edman degradation protein sequencer, the N-terminal amino acid sequence of the subunit was determined to be SKIRIGIVGY.

### Gene sequencing

To identify the *meso*-DAPDH gene in the chromosome of *U. thermosphaeicus*, *in vitro *cloning was performed as described in materials and methods with information of N-terminal amino acid sequence. Sequencing of the fragment revealed a single 981-bp open reading frame encoding 326 amino acids (accession number: AB636161), and the deduced N-terminal amino acid sequence was identical to that obtained by protein sequencing. An NCBI BLAST search revealed the amino acid sequence of *U. thermosphaeicus meso*-DAPDH to be highly homologous with the sequence of *meso*-DAPDH from *B. sphaericus *(80%), *Bacillus *sp. B14905 (79%), *Cl. thermocellum *ATCC 27405 (64%) and with those from the putative *meso*-DAPDHs from *Lysinibacillus fusiformis *ZC1 (80%), *L. sphaericus *C3-41 (80%), *Cl. tetani *E88 (66%) and *Herminiimonas arsenicoxydans *(66%).

### Purification and characterization of recombinant *meso*-DAPDH expressed in *E. coli*

After transformation of *E. coli *Rosetta (DE3) cells with pET101/DAPDH, which harbored the recombinant *meso-*DAPDH gene, a high level of *meso-*DAPDH production was found in the crude extract of the transformants. The recombinant *meso-*DAPDH was easily purified about 2.4-fold, with an overall yield of about 53.1%, by successive heat-treatment and Chelating Sepharose Fast Flow column chromatography steps. With this procedure, we obtained 54.6 mg of the purified protein from 2.38 g (wet weight) of *E. coli *cells (Table 2).

**Table 2:** Purification of recombinant NADP-dependent *meso-*DAPDH from *E. coli*

**Purification step**^**b**^	Total protein	Total activity	Specific activity	Yield
	mg	units	units/mg	%
Crude extract	250	796	3.18	100
Heat-treatment	198	694	3.51	87.1
Chelating Sepharose™ Fast Flow column	54.6	423	7.75	53.1

b) The cells were obtained from a 500 ml-medium

The maximum activity of the purified recombinant *meso*-DAPDH was detected at 65°C in carbonate-KOH buffer at pH 10.5. Full activity was retained after incubation at 60°C for 30 min, but activity was completely lost after incubation at 70°C for 30 min. There was no significant difference in the enzyme stability or the molecular mass between the purified native *meso*-DAPDH and the purified recombinant *meso*-DAPDH.

## Discussion

Misono et al. (1979); (1986a) previously described the relatively wide distribution of *meso-*DAPDHs among mesophilic bacteria, including *B. sphaericus*, *Brevibacterium *sp., *C. glutamicum *and *Proteus vulgaris*. The presence of *meso-*DAPDH in thermophile has not been found until the recent report on the enzyme from an anerobic *Cl. thermocellum *(Hudson et al. 2011). We found one enzyme producer in many aerobic thermophiles by extensive screening. The thermophile was identicafied to be *U. thermosphaeicus *strain A1. This is the first producer of *meso-*DAPDH in aerobic thermophile.

As expected, *meso*-DAPDH purified from *U. thermosphaericus *is much more thermostable than its counterparts from mesophiles, such as *B. sphaericus *(Misono and Soda 1980) and *C. glutamicum *(Misono et al 1986a): the *U. thermosphaericus *enzyme showed almost no loss of activity after incubation for 30 min at temperatures up to 60°C, whereas the mesophilic enzymes lost their activity within 10 min at temperatures above 48°C. There is no report with respect to the thermostability of the *Cl. thermocellum *enzyme (Hudson et al. 2011). In addition, the *U. thermosphaericus *enzyme is highly stable over wider range of pHs (no loss of activity at pH 5.0 to 11.0 after incubation for 30 min at 50°C) than the mesophilic enzymes (*Brevibacterium *sp. enzyme: stable at pH 7.0-9.0 after 10 min at 40°C; *C. glutamicum *enzyme: stable at pH 6.5-7.0 after 10 min at 48°C) (Misono et al 1986a, b). The higher stability of the *U. thermosphaericus *enzyme under the various conditions tested suggests it would be more useful and easier to obtain in a highly purified form needed for bioprocesses. Still, the dimeric structure of the *U. thermosphaericus meso-*DAPDH and its narrow substrate specificity for *meso-*DAP and NADP indicates that it is not very different against mesophilic counterparts.

We also succeeded in cloning and sequencing the *meso-*DAPDH gene from *U. thermosphaericus*, which enabled us to greatly enhance production of the enzyme in recombinant *E. coli *cells. The increased production was in part due to the much more effective method of purification, which had a yield of 53.1% from the recombinant cells, as compared to 4.69% from *U. thermosphaericus *cells. Vedha et al. (2006) used a protein engineering method to create a highly stereoselective NADP-dependent D-amino acid dehydrogenase with broad substrate specificity from *C. glutamicum meso*-DAPDH and showed its application for one-step synthesis of D-amino acids from their oxo analogs. The sequence alignment of *meso-*DAPDHs of *U. thermosphaericus *and other four bacterial strains indicates that all five amino acids (Q150, D154, T169, R195 and H244) in *C. glutamicum *enzyme mutated for the creation of D-amino acid dehydrogenase are perfectly conserved in the sequences of other bacterial enzymes containing *U. thermosphaericus *enzyme (Figure 1). The *U. thermosphaericus meso*-DAPDH is much more stable than the *C. glutamicum meso*-DAPDH just mentioned. Thus, the *U. thermosphaericus *enzyme has the potential for use in the creation of a more stable stereoselective NADP-linked D-amino acid dehydrogenase. In addition to altering its substrate specificity, we are planning to create a novel stable NAD-linked D-amino acid dehydrogenase by designing its coenzyme specificity based on the sequence data from *U. thermosphaericus meso*-DAPDH and detailed information about the 3D-structure (Scapin et al 1996) and active sites (Scapin et al. 1998) of the *C. glutamicum *enzyme. Such an NAD or NAD(P)-dependent D-amino acid dehydrogenase could be highly useful in a variety of bioprocesses for the production and sensing of D-amino acids and their analogs. Furthermore, we have already identified some putative *meso-*DAPDH genes in hyperthermophilic bacteria like *Thermotoga *species by sequence database, and the functional analyses of *meso-*DAPDH gene homolog in *Thermotoga *species are now under investigation.

## Competing interests

The authors declare that they have no competing interests.

## References

[B1] Bradford MM A rapid and sensitive method for the quantitation of microgram quantities of protein utilizing the principle of protein-dye binding Anal Biochem 1976 72 248 254 10.1016/0003-2697(76)90527-3 942051

[B2] Davis BJ Disc electrophoresis - II method and application to human Ann N Y Acad Sci 1964 121 404 427 14240539 10.1111/j.1749-6632.1964.tb14213.x

[B3] Fortina MG Pukall R Schumann P Mora D Parini C Manachini PL Stackebrandt E *Ureibacillus *gen. nov., a new genus to accomodate *Bacillus thermosphaericus *(Andersson et al. 1995), emendation of *Ureibacillus thermosphaericus *and description of *Ureibacillus terrenus *sp. nov Int J Syst Evol Microbiol 2001 51 447 455 11321090 10.1099/00207713-51-2-447

[B4] Hudson AO Klartag A Gilvarg C Dobson RC Marques FG Leustek T Dual diaminopimelate biosynthesis pathways in *Bacteroides fragilis *and *Clostridium thermocellum* Biochim Biophys Acta 2011 1814 1162 1168 21616177 10.1016/j.bbapap.2011.04.019

[B5] Ishino S Mizukami T Yamaguchi K Katsumata R Araki K Cloning and sequencing of the *meso*-diaminopimelate-D-dehydrogenase (ddh) gene of *Corynebacterium glutamicum* Agric Biol Chem 1988 52 2903 2909 10.1271/bbb1961.52.2903

[B6] Laemmli UK Cleavage of structural proteins during the assembly of the head of bacteriophage T4 Nature 1970 227 680 685 10.1038/227680a0 5432063

[B7] Larkin MA Blackshields G Brown NP Chenna R McGettigan PA McWilliam H Valentin F Wallace IM Wilm A Lopez R Thompson JD Gibson TJ Higgins DG Clustal W and Clustal X version 2.0 Bioinformatics 2007 23 2947 2948 10.1093/bioinformatics/btm404 17846036

[B8] Misono H Togawa H Yamamoto T Soda K *meso*-α,ε-Diaminopimelate D-dehydrogenase: distribution and the reaction product J Bacteriol 1979 137 22 27 762012 10.1128/jb.137.1.22-27.1979 PMC218413

[B9] Misono H Ogasawara M Nagasaki S Characterization of *meso*-diaminopimelate dehydrogenase from *Corynebacterium glutamicum *and its distribution in bacteria Agric Biol Chem 1986 50 2729 2734 10.1271/bbb1961.50.2729

[B10] Misono H Ogasawara M Nagasaki S Purification and properties of *meso*-diaminopimelate dehydrogenase from *Brevibacterium *sp Agric Biol Chem 1986 50 1329 1330 10.1271/bbb1961.50.1329

[B11] Misono H Soda K Properties of *meso*-α,ε-diaminopimelate D-dehydrogenase from *Bacillus sphaericus* J Biol Chem 1980 255 10599 10605 7430138

[B12] Ohshima T Ishida M A large-scale preparative electrophoretic method for the purification of pyridine nucleotide-linked dehydrogenases Protein Expr Purif 1992 2 121 125 1422215

[B13] Reddy SG Scapin G Blanchard JS Expression, purification, and crystallization of *meso*-diaminopimelate dehydrogenase from *Corynebacterium glutamicum* Proteins 1996 4 514 516 10.1002/prot.12 8865347

[B14] Sakamoto S Seki M Nagata S Misono H Cloning, sequencing, and expression of the *meso*-diaminopimelate dehydrogenase gene from *Bacillus sphaericus* J Mol Catal B Enzym 2001 12 85 92 10.1016/S1381-1177(00)00207-1

[B15] Scapin G Reddy SG Blanchard JS Three-dimensional structure of *meso*-diaminopimelic acid dehydrogenase from *Corynebacterium glutamicum* Biochemistry 1996 35 13540 13551 10.1021/bi961628i 8885833

[B16] Scapin G Cirilli M Reddy SG Blanchard JS Substrate and inhibitor binding sites in *Corynebacterium glutamicum *diaminopimelate dehydrogenase Biochemistry 1998 37 3278 3285 10.1021/bi9727949 9521647

[B17] Vedha PK Gunawardana M Rozzell JD Novick SJ Creation of a broad-range and highly stereoselective D-amino acid dehydrogenase for the one-step synthesis of D-amino acids J Am Chem Soc 2006 128 10923 10929 10.1021/ja0603960 16910688 PMC2533268

